# Caveolar Fatty Acids and Acylation of Caveolin-1

**DOI:** 10.1371/journal.pone.0060884

**Published:** 2013-04-11

**Authors:** Qian Cai, Ling Guo, Haiqing Gao, Xiang-An Li

**Affiliations:** 1 Department of Pediatrics, University of Kentucky College of Medicine, Lexington, Kentucky, United States of America; 2 Department of Geriatrics, Qi-Lu Hospital of Shandong University, Jinan, Shandong, P. R. China; Biological Research Centre of the Hungarian Academy of Sciences, Hungary

## Abstract

**Purpose:**

Caveolae are cholesterol and sphingolipids rich subcellular domains on plasma membrane. Caveolae contain a variety of signaling proteins which provide platforms for signaling transduction. In addition to enriched with cholesterol and sphingolipids, caveolae also contain a variety of fatty acids. It has been well-established that acylation of protein plays a pivotal role in subcellular location including targeting to caveolae. However, the fatty acid compositions of caveolae and the type of acylation of caveolar proteins remain largely unknown. In this study, we investigated the fatty acids in caveolae and caveolin-1 bound fatty acids.

**Methods:**

Caveolae were isolated from Chinese hamster ovary (CHO) cells. The caveolar fatty acids were extracted with Folch reagent, methyl esterificated with BF3, and analyzed by gas chromatograph-mass spectrometer (GC/MS). The caveolin-1bound fatty acids were immunoprecipitated by anti-caveolin-1 IgG and analyzed with GC/MS.

**Results:**

In contrast to the whole CHO cell lysate which contained a variety of fatty acids, caveolae mainly contained three types of fatty acids, 0.48 µg palmitic acid, 0.61 µg stearic acid and 0.83 µg oleic acid/caveolae preparation/5×10^7^ cells. Unexpectedly, GC/MS analysis indicated that caveolin-1 was not acylated by myristic acid; instead, it was acylated by palmitic acid and stearic acid.

**Conclusion:**

Caveolae contained a special set of fatty acids, highly enriched with saturated fatty acids, and caveolin-1 was acylated by palmitic acid and stearic acid. The unique fatty acid compositions of caveolae and acylation of caveolin-1 may be important for caveolae formation and for maintaining the function of caveolae.

## Introduction

Caveolae are cholesterol and sphingomyelin-rich plasma membrane microdomains presented in most types of mammalian cells and tissues[1]–[5]_ENREF_13. Caveolae were originally identified as 50–100 nm flask-shaped, non-clathrin-coated invagination of the plasma membrane and found to be involved in endocytosis [6] and potocytosis [7]. However, later studies revealed that these microdomains concentrate a variety of signaling molecules which provide a platform for signal transduction[8]–[12]. Thus, changes in the components of caveolae may have a profound effect on cellular functions. Caveolin-1, a 22-kDa protein, is the principal structural component of caveolae and a determinant for caveolae formation [13], [14]. A deficiency of caveolin-1 in mice eliminated caveolae, which subsequently impaired nitric oxide and calcium signaling in the cardiovascular system, causing aberrations in endothelium-dependent relaxation, contractility, and maintenance of myogenic tone [15], [16].

In addition to cholesterol and sphingomyelin, caveolae also contain a variety of fatty acids. While it is generally believed that covalent attachment of myristic and/or palmitic acid occurs on a wide variety of cellular proteins and the acylation of protein is critical for membrane targeting [17], the fatty acid compositions of caveolae, the fatty acids bound to caveolin-1, and the effect of acylation of caveolin-1 on caveolin-1 targeting to caveolae remain poorly understood. In this study, we used gas chromatography/mass spectrometry (GC/MS) to identify and quantify the fatty acid compositions of caveolae and fatty acids covalently bound to caveolin-1 in Chinese hamster ovary (CHO) cells, a cell system with high caveolin-1 expression. Our results revealed that caveolae contain a limited subset of fatty acids, highly enriched with saturated fatty acids, which is quite different from the fatty acid compositions in whole cells. We further demonstrated that the primary fatty acid associated with caveolin-1 is stearic acid, not myristic acid as previous speculated.

## Materials and Methods

### Materials

Bovine serum albumin (BSA, fatty acid free) and general chemical reagents were obtained from Sigma. BF3 reagent, mixture of standard fatty acids and Omegawax 250 capillary column were from Sigma/Supelco. Ham’s F-12 medium and fetal bovine serum (FBS) were from Invitrogen. Opti-Prep was from Life Technologies. The protein A-Sepharose beads were from GE Health. The anti-caveolin-1 IgG was from Transduction Laboratories; non-immune rabbit IgG was from Jackson laboratory.

### Preparation of Whole Cell Lysate

Chinese hamster ovary (CHO) cells were cultured in Ham’s F-12 medium containing 5% FBS, 2 mmol/L L-glutamine, 100 U/ml penicillin and 100 µg/ml streptomycin to 90% confluency in 10 cm culture dish. The cells were washed 5 times with 10 ml of cold PBS and dissolved in 1 ml of MBST/OG (25 mM MES, 150 mM NaCl, 1% Triton-100, 60 mM octyglucopyranoside, pH 6.7) for 30 min on ice.

### Preparation of Caveolae

Caveolae, cytosol, plasma membrane, internal membranes and post nuclear supernatant fractions were isolated using a widely used Opti-Prep method as described previously [18]_ENREF_19. This detergent-free method generates a highly purified fraction of caveolae that is enriched in caveolin-1 and free of bulk plasma membrane markers.

### Quantification of Fatty Acids Associated with Caveolin-1

The CHO cell lysates were incubated with anti-caveolin-1 IgG for 18 h at 4°C and then protein A beads for an additional 2 h at 4°C. The beads were collected by centrifugation, washed five times in high salt buffer (500 mM NaCl). For quantification of fatty acids, the beads were incubated with 1M NaOH overnight, and then neutralized by 1M HCL, extracted with Folch reagent and analyzed by GC/MS. Non-immune rabbit IgG was used as negative control.

### Quantification of Fatty Acids with GC/MS

Quantification of fatty acids was conducted as we previously described [19]. Briefly, the lipids in samples were extracted with Folch/BHT reagent [20] and lower phase of the extract was collected and dried under N_2_. Fifty microlitter of tricosanoic acid (23∶0) (5 mg/ml chloroform) was added to extract as an internal standard. Tissue total lipids were methyl esterified with BF3/Methanol (10%). The fatty acid methyl ester was analyzed using a gas chromatography system, Agilent 6890 GC G2579A system (Agilent Palo Alto, CA) equipped with an Omegawax 250 capillary column. An Agilent 5973 mass selective detector was used to identify target peaks and a flame ionization detector (FID) was used to quantify fatty acids.

### Western Blot

Western blot was performed as described previously [21].

### Characterization of Fatty Acid Esterification of Caveolin-1

CHO cells were cultured in Ham’s F-12 medium containing 5% FBS, 2 mmol/L L-glutamine, 100 U/ml penicillin and 100 µg/ml streptomycin to 80% confluency in 10 cm culture dish. The cells were starved 18 h in Ham’s F-12 medium containing 1% BSA and then labeled with 2.5 mCi of ^3^H-palmitic acid or 25 µCi of ^14^C-stearic acid for 3 h at 37°C in the presence/absence of 30 times of non-labeled palmitic acid (16∶0), stearic acid (18∶0) or oleic acid (18∶1). The cells were dissolved in MBST/OG buffer and immunoprecipitated with anti-caveolin-1/protein A. Non-immune rabbit IgG was used as negative control. The immunoprecipitated caveolin-1 was separated by SDS-PAGE and transferred to PVDF membrane. The fatty acid esterification of caveolin-1 was detected by autoradiogram at −80°C for 6 weeks using a Kodak MS film.

## Results

**Figure 1:**
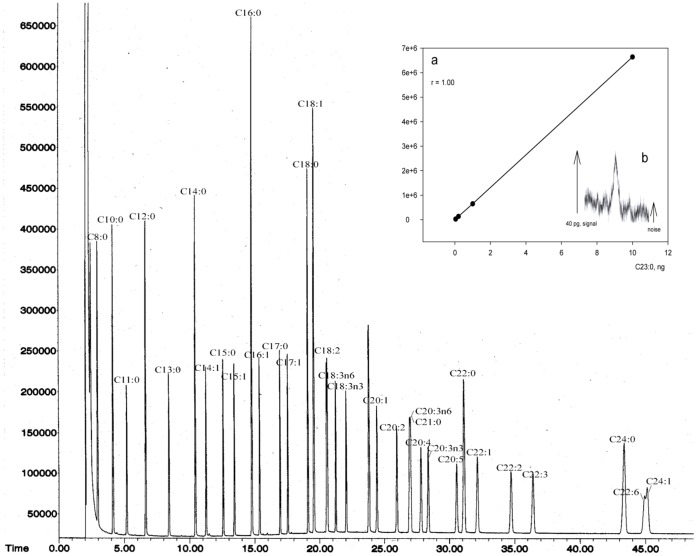
Separation of fatty acid methyl esters and determination of the sensitivity and linear range of GC/MS method. A mixture of standard fatty acid esters was subjected to GC/MS equipped with Omegawax 250 capillary column. Fatty acids were identified by mass spectrometer and quantified by FID. The measurement of fatty acid methyl esters with GC/MS was of high sensitivity, up to 40 pg of tricosanoic methyl ester, and had a wide linear range, up to 10 ng (inner figure, a and b).

GC/MS is a powerful tool for qualitative and quantitative analysis of fatty acids. Here, we selected Omegawax 250 capillary column and tested its efficiency in fatty acid separation using a mixture of standard fatty acid methyl esters. As shown in Figure 1, GC/MS with Omegawax 250 column separated all the fatty acids presented in animal tissue. The measurement of fatty acid methyl esters was of high sensitivity, up to 40 pg of tricosanoic methyl ester, and had a wide linear range, up to 10 ng (Figure 1, inner figure).

**Figure 2:**
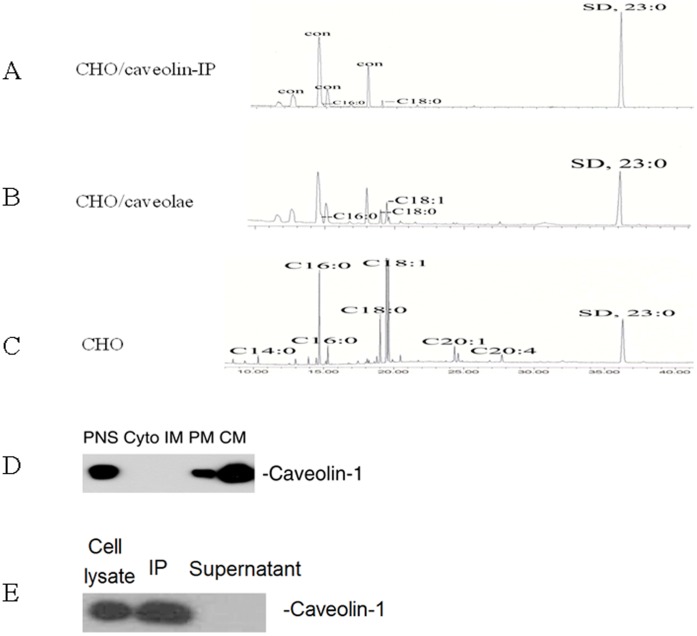
Quantification of fatty acids bound to caveolin-1, associated with caveolae, and present in CHO cells. The CHO cells were cultured in Ham’s F-12 medium to 90% confluency. After washed with PBS, they were dissolved in MBST/OG on ice. Post nuclear supernatant (PNS), cytosol (Cyto), internal membranes (IM), plasma membrane (PM), and caveolae (CM) were isolated with Opti-Prep method. Total fatty acids were extracted from each sample with Folch reagent, methyl esterified with BF3, and then subjected to GC/MS equipped with Omegawax 250 capillary column. Fatty acids bound to caveolin-1 (A), associated with caveolae (B), and present in CHO cells (C) were identified by MS. (D) Isolation of caveolae from CHO cells. Subcellular fractions were isolated with Opti-Prep method and subjected to Western blot using antibody against caveolin-1. (E) Immunoprecipitation of caveolin-1. The CHO cell lysates were immunoprecipitated with anti-caveolin-1 IgG/protein A-Sepharose beads and detected by Western blot using antibody against caveolin-1. The experiments were repeated three times with triplicate measurements. Quantitative analysis of the data is shown in Figure 3.

**Figure 3:**
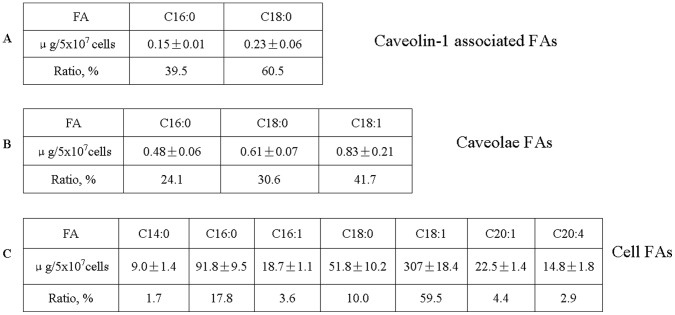
Quantification of fatty acids bound to caveolin-1, associated with caveolae, and present in CHO cells. The CHO cells were cultured in Ham’s F-12 medium to 90% confluency. Caveolae were isolated with Opti-Prep method and caveolin-1 was purified by immunoprecipitation. Total fatty acids were extracted from each sample with Folch reagent, methyl esterified with BF3, and then subjected to GC/MS equipped with Omegawax 250 capillary column. Fatty acids bound to caveolin-1 (A), associated with caveolae (B), and present in CHO cells (C) were quantified with FID. The experiments were repeated three times with triplicate measurements. Data are presented as Mean ± SD.

We employed CHO cells to study the fatty acid compositions of caveolae and fatty acids associated with caveolin-1. First, we measured the fatty acids in CHO cells. The CHO cells were cultured in Ham’s F-12 medium to 90% confluency. Total fatty acids were extracted and analyzed with GC/MS (Figure 2C). CHO cells contained a variety of fatty acids, including both saturated and unsaturated fatty acids (Figure 3C). Of all these fatty acids, oleic acid (C18∶1, 307±18.4 µg/5×10^7^ cells) accounted for the greatest part as high as 59.5%, followed by palmitic acid (C16∶0, 91.8±9.5 µg/5×10^7^ cells; 17.8%), stearic acid (C18∶0, 51.8±10.2 µg/5×10^7^ cells; 10%), gadoleic acid (C20∶1, 22.5±1.4 µg/5×10^7^ cells; 4.4%), palmitoleic acid (C16∶1, 18.7±1.1 µg/5×10^7^ cells; 3.6%), arachidonic acid (C20∶4, 14.8±1.8 µg/5×10^7^ cells; 2.9%) and myristic acid (C14∶0, 9.0±1.4 µg/5×10^7^ cells; 1.7%).

Then we isolated caveolae from CHO cells by using Opti-Prep method [22]. We isolated post nuclear supernatant (PNS), cytosol (Cyto), internal membrane (IM), plasma membrane (PM) and caveolae (CM) fractions. Western blot analysis of the subcellular fractions showed that caveolin-1 was highly enriched in CM fraction, indicating successful isolation of caveolae (Figure 2D). Then we extracted fatty acids from caveolae and quantified the fatty acid compositions with GC/MS (Figures 2B and 3B). We found that caveolae contain a limited subset of total fatty acids in cells, mainly palmitic acid (C16∶0, 0.48±0.06 µg/5×10^7^ cells; 24%), stearic acid (C18∶0, 0.61±0.07 µg/5×10^7^ cells; 30%) and oleic acid (C18∶1, 0.83±0.21 µg/5×10^7^ cells; 40%) (Figure 3B).

Next, we isolated caveolin-1 by immunoprecipitation and quantified fatty acids bound to caveolin-1. Western blot analysis indicated that caveolin-1 was completely immunoprecipitated by anti-Caveolin-1 IgG (Figure 2E), but not by non-immune rabbit IgG (data not shown), indicating successful isolation of caveolin-1. It is generally believed that plasma membrane proteins are acylated by myristic acid and/or by palmitic acid [17]. Surprisingly, we found that the primary fatty acid associated with caveolin-1 is stearic acid. About 60% of the associated fatty acids is stearic acid (C18∶0, 0.23±0.06 µg/5×10^7^ cells) and 40% of the associated fatty acids is palmitic acid (C16∶0, 0.15±0.01 µg/5×10^7^ cells) (Figure 3A). No myristic acid was found to be associated with caveolin-1. Of note, oleic acid was found to be the most abundant fatty acid in caveolae but it was not detected in caveolin-1 bound fraction (Figures 2 and 3). MS identified a number of peaks as non-fatty acid contaminant, which were marked as “con” on Figure 2A.

**Figure 4:**
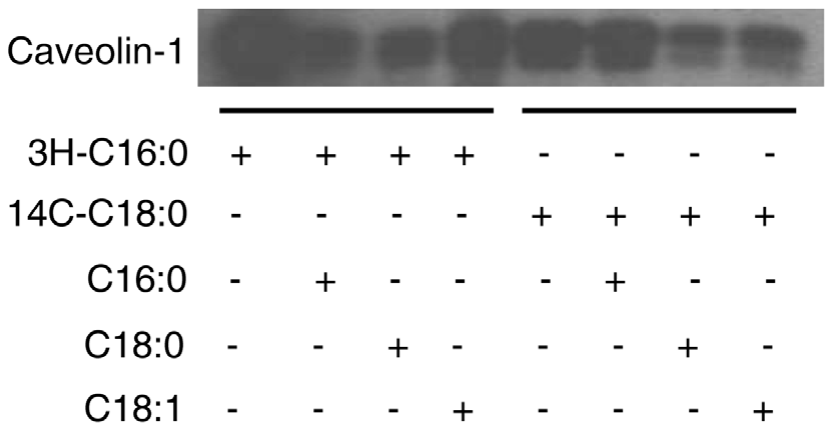
Acylation of caveolin-1. CHO cells were cultured in Ham’s F-12 complete medium to 80% confluency. The cells were starved and then labeled with 2.5 mCi of ^3^H-palmitic acid or 25 µCi of ^14^C-stearic acid for 3 h at 37°C in the presence/absence of 30 times of non-labeled palmitic acid (C16∶0), stearic acid (C18∶0) or oleic acid (C18∶1). The cells were harvested in MBST/OG buffer and immunoprecipitated with anti-acveolin-1 IgG/protein A. The immunoprecipitated caveolin-1 was separated by SDS-PAGE, transferred to a membrane and the fatty acid associated with caveolin-1 was detected with autoradiogram. The experiments were repeated three times, and representative data are shown.

To further characterize the fatty acids bound to caveolin-1, we utilized radioisotope labeled fatty acids. CHO cells were cultured in Ham’s F-12 medium to 80% confluency and starved for 18 h. Then the cells were labeled with 2.5 mCi of ^3^H-palmitic acid or 25 µCi of ^14^C-stearic acid for 3 h at 37°C in the presence/absence of 30 times of non-labeled palmitic acid (C16∶0), stearic acid (C18∶0) or oleic acid (C18∶1). In cells labeled with ^3^H-palmitic acid, we detected ^3^H-palmitic acid bound to caveolin-1, indicating that palmitic acid binds to caveolin-1directly (Figure 4). In the presence of excessive amount of non-labeled palmitic acid, non-labeled palmitic acid effectively blocked the binding of ^3^H-palmitic acid to caveolin-1. Excessive amount of non-labeled stearic acid also effectively blocked the binding of ^3^H-palmitic acid to caveolin-1. However, excessive amount of non-labeled oleic acid only moderately blocked the binding of ^3^H-palmitic acid to caveolin-1. Likewise, in the cells labeled with ^14^ C-stearic acid, we detected ^14^C-stearic acid bound to caveolin-1, indicating that stearic acid binds to caveolin-1directly. Excessive amount of non-labeled stearic acid could effectively inhibit the binding of ^14^C-stearic acid to caveolin-1. Excessive amount of non-labeled oleic acid or palmitic acid blocked the binding of ^14^C-stearic acid to caveolin-1 but with much less efficiency compared to stearic acid. These data demonstrated that palmitic acid and stearic acid are the main fatty acids binding to caveolin-1.

**Figure 5:**
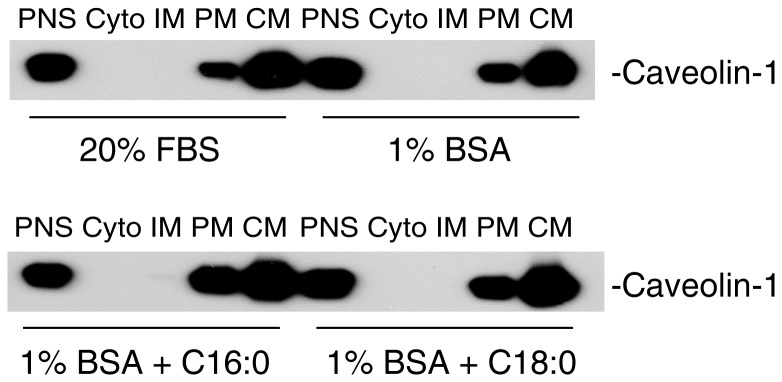
Effect of fatty acids on caveolin-1 subcellular location. CHO cells were cultured in Ham’s F-12 complete medium to 80% confluency. The cells were culture for 24 h with Ham’s F-12 containing 20% FBS, 1% BSA (fatty acid free), 1% BSA plus 200 µg/ml palmitic acid or 1% BSA plus 200 µg/ml stearic acid. The subcellular fractions were isolated with Opti-Prep method and subjected to Western blot using antibody against caveolin-1. The experiments were repeated two times and representative data are shown.

Acylation of protein has been shown to affect the subcellular targeting of the protein [17]. To test whether acylation of caveolin-1 affects caveolin-1subcellular location, we cultured CHO cells in the presence/absence of palmitic acid or stearic acid. As shown in Figure 5, the targeting of caveolin-1 to caveolae was not affected by the presence of palmitic acid or stearic acid.

## Discussion

GC/MS offers high chromatographic resolution, wide linear range, high sensitivity, and excellent reproducibility [19]. In this study, we employed GC/MS with a polar column to quantify fatty acids in caveolae and associated with caveolin-1. We found that caveolae contain a limited subset of the fatty acids in cells, including palmitic acid, stearic acid and oleic acid. Unexpectedly, we found that caveolin-1 is acylated by stearic acid and palmitic acid, not by myristic acid, and the acylation of caveolin-1 does not affect caveolin-1 targeting to caveolae.

Based on the character that caveolae are insoluble in detergent Triton x-100, Gafencu et al. isolated caveolae from aortic endothelial cells and measured the fatty acid compositions of caveolae with gas chromatography [23]. The authors found that there are significant increases in the ratios of palmitic acid, stearic acid and oleic acid in caveolae. Using MCF7 cells (a breast cancer cell line), Hout et al also observed significant increases in the ratios of palmitic acid, stearic acid and oleic acid in caveolae [24]. However both studies did not quantify the absolute amount of fatty acid in caveolae. In this study, we used the Opti-Prep method to isolate caveolae and quantify the amount of fatty acid in caveolae in CHO cells using tricosanoic acid as an internal standard. Opti-Prep is a detergent-free method which can isolate highly purified caveolae [18].We found that caveolae indeed contained high levels of palmitic acid, stearic acid and oleic acid. Thus evidence from three different types of cells and two independent approaches pointed out that caveolae are enriched with saturated fatty acids compare to the fatty acid compositions in whole cells and the enrichment of saturated fatty acids in caveolae is likely a character of caveolae, regardless of cell types.

Acylation of protein has been demonstrated to be a principle posttranslational modification of protein, which plays a pivotal role in protein subcellular targeting and cellular function [17]. For example, palmitoylation and myristoylation can both influence the localization of eNOS to caveolae [25], [26]. To determine the acylation of caveolin-1, we purified caveolin-1 by immunoprecipitation and quantified fatty acid with GC/MS. We unexpectedly found that caveolin-1 is acylated by stearic acid and palmitic acid, not by myristic acid. To the best of our knowledge, this was the first direct characterization and quantification of fatty acid associated with caveolin-1.

To further characterize the acylation of caveolin-1, we utilized ^3^H-palmitic acid and ^14^C-stearic acid combined with competitive inhibition by non-labeled fatty acids. We found that acylation of caveolin-1 by ^3^H-palmitic acid is effectively inhibited by non-labeled palmitic acid or stearic acid. However, acylation of caveolin-1 by ^14^C-stearic acid is only effectively inhibited by non-labeled stearic acid, but not by palmitic acid. These findings indicate that some of the acylation sites on caveolin-1 can only be acylated by stearic acid. Although we did not detect any existing oleic acid binding to caveolin-1 by GC/MS, we found that non-labeled oleic acid moderately inhibits ^14^C-stearic acid and ^3^H-palmitic acid binding to caveolin-1. We speculate that oleic acid may hinder the binding of stearic acid and palmitic acid to caveolin-1.

As acylation of protein has been shown to affect the subcellular targeting of the protein [17]. We tested whether acylation of caveolin-1 by stearic acid or palmitic acid affects caveolin-1subcellular location. We did not observe an effect of stearic acid or palmitic acid on the targeting of caveolin-1 to caveolae. This is in agreement with an early report showing that palmitoylation of caveolin-1 is not required for caveolae targeting [27]. However, available evidence indicates that acylation of caveolin-1 is critical for the function of caveolin-1. Utilizing acylation site mutant caveolin-1 (C133S, C143S, and C156S), Galbiati et al demonstrated that lack of acylation, caveolin-1 was unable to bind with G-protein G_i1α_ in caveolae [28]; Lee et al showed that palmitoylation of caveolin-1 at a single site (C156) is required for coupling caveolin-1 to the c-Src tyrosine kinase, which is critical for phosphorylation of caveolin-1 [29].

In summary, our study demonstrates that caveolae contain a special set of fatty acids, highly enriched with saturated fatty acids, and caveolin-1 is acylated by palmitic acid and stearic acid. The unique fatty acid compositions of caveolae and acylation of caveolin-1 may be important for caveolae formation and for maintaining the function of caveolae.

## References

[1] Sowa G (2012) Caveolae, caveolins, cavins, and endothelial cell function: new insights. Front Physiol 2: 120. 22232608 10.3389/fphys.2011.00120 PMC3252561

[2] Palade GE (1953) Fine structure of blood capillaries. Journal of Applied Physics 24: 1424.

[3] Palade GE (1961) Blood capillaries of the heart and other organs. Circulation 24: 368–384. 13732173 10.1161/01.cir.24.2.368

[4] Palade GE (1968) Structural modulation of plasmalemmal vesicles. Journal of Cell Biology 37: 633–649. 11905197 10.1083/jcb.37.3.633 PMC2107438

[5] Yamada E (1953) The fine structure of the gall bladder epithelium of the mouse. Journal of Biophysical Biochemistry and Cytology 1: 445–458. 10.1083/jcb.1.5.445 PMC2229656 13263332

[6] Schnitzer JE, Oh P, McIntosh DP (1996) Role of GTP hydrolysis in fission of caveolae directly from plasma membranes [published erratum appears in Science 1996 Nov 15;274(5290): 1069]. Science 274: 239–242. 8824187 10.1126/science.274.5285.239

[7] Anderson RG, Kamen BA, Rothberg KG, Lacey SW (1992) Potocytosis: sequestration and transport of small molecules by caveolae. Science 255: 410–411. 1310359 10.1126/science.1310359

[8] Allen JA, Halverson-Tamboli RA, Rasenick MM (2007) Lipid raft microdomains and neurotransmitter signalling. Nat Rev Neurosci 8: 128–140. 17195035 10.1038/nrn2059

[9] Parton RG, Simons K (2007) The multiple faces of caveolae. Nat Rev Mol Cell Biol 8: 185–194. 17318224 10.1038/nrm2122

[10] Insel PA, Patel HH (2009) Membrane rafts and caveolae in cardiovascular signaling. Curr Opin Nephrol Hypertens 18: 50–56. 19077689 10.1097/MNH.0b013e3283186f82 PMC2757134

[11] Patel HH, Murray F, Insel PA (2008) Caveolae as organizers of pharmacologically relevant signal transduction molecules. Annu Rev Pharmacol Toxicol 48: 359–391. 17914930 10.1146/annurev.pharmtox.48.121506.124841 PMC3083858

[12] Li XA, Everson WV, Smart EJ (2005) Caveolae, lipid rafts, and vascular disease. Trends Cardiovasc Med 15: 92–96. 16039968 10.1016/j.tcm.2005.04.001

[13] Jin Y, Lee SJ, Minshall RD, Choi AM (2011) Caveolin-1: a critical regulator of lung injury. Am J Physiol Lung Cell Mol Physiol 300: L151–160. 21097526 10.1152/ajplung.00170.2010 PMC4380484

[14] Rothberg KG, Heuser JE, Donzell WC, Ying YS, Glenney JR, et al (1992) Caveolin, a protein component of caveolae membrane coats. Cell 68: 673–682. 1739974 10.1016/0092-8674(92)90143-z

[15] Drab M, Verkade P, Elger M, Kasper M, Lohn M, et al (2001) Loss of caveolae, vascular dysfunction, and pulmonary defects in caveolin-1 gene-disrupted mice. Science 293: 2449–2452. 11498544 10.1126/science.1062688

[16] Razani B, Engelman JA, Wang XB, Schubert W, Zhang XL, et al (2001) Caveolin-1 Null Mice Are Viable but Show Evidence of Hyperproliferative and Vascular Abnormalities. J Biol Chem 276: 38121–38138. 11457855 10.1074/jbc.M105408200

[17] Resh MD (1999) Fatty acylation of proteins: new insights into membrane targeting of myristoylated and palmitoylated proteins. Biochim Biophys Acta 1451: 1–16. 10446384 10.1016/s0167-4889(99)00075-0

[18] Smart EJ, Ying YS, Mineo C, Anderson RG (1995) A detergent-free method for purifying caveolae membrane from tissue culture cells. Proc Natl Acad Sci U S A 92: 10104–10108. 7479734 10.1073/pnas.92.22.10104 PMC40744

[19] Liu J, Sabeva NS, Bhatnagar S, Li XA, Pujol A, et al (2010) ABCD2 is abundant in adipose tissue and opposes the accumulation of dietary erucic acid (C22:1) in fat. J Lipid Res 51: 162–168. 19556607 10.1194/jlr.M900237-JLR200 PMC2789776

[20] Folch J, Lees M, Stanley GHS (1957) A Simple Method for the Isolation and Purification of Total Lipides from Animal Tissues. Journal of Biological Chemistry 226: 497–509. 13428781

[21] Li XA, Guo L, Asmis R, Nikolova-Karakashian M, Smart EJ (2006) Scavenger receptor BI prevents nitric oxide-induced cytotoxicity and endotoxin-induced death. Circ Res 98: e60–65. 16574909 10.1161/01.RES.0000219310.00308.10 PMC10961161

[22] Uittenbogaard A, Ying Y, Smart EJ (1998) Characterization of a cytosolic heat-shock protein-caveolin chaperone complex. Involvement in cholesterol trafficking. J Biol Chem 273: 6525–6532. 9497388 10.1074/jbc.273.11.6525

[23] Gafencu A, Stanescu M, Toderici AM, Heltianu C, Simionescu M (1998) Protein and fatty acid composition of caveolae from apical plasmalemma of aortic endothelial cells. Cell Tissue Res 293: 101–110. 9634602 10.1007/s004410051102

[24] Huot PS, Sarkar B, Ma DW (2010) Conjugated linoleic acid alters caveolae phospholipid fatty acid composition and decreases caveolin-1 expression in MCF-7 breast cancer cells. Nutr Res 30: 179–185. 20417878 10.1016/j.nutres.2010.02.003

[25] Robinson LJ, Busconi L, Michel T (1995) Agonist-modulated palmitoylation of endothelial nitric oxide synthase. J Biol Chem 270: 995–998. 7530714 10.1074/jbc.270.3.995

[26] Bernatchez P, Sharma A, Bauer PM, Marin E, Sessa WC (2011) A noninhibitory mutant of the caveolin-1 scaffolding domain enhances eNOS-derived NO synthesis and vasodilation in mice. J Clin Invest 121: 3747–3755. 21804187 10.1172/JCI44778 PMC3163946

[27] Dietzen D, Hastings W, Lublin D (1995) Caveolin is palmitoylated on multiple cysteine residues: palmitoylation is not necessary for localization of caveolin to caveolae. J Biol Chem 270: 6838–6842. 7896831 10.1074/jbc.270.12.6838

[28] Galbiati F, Volonte D, Meani D, Milligan G, Lublin DM, et al (1999) The dually acylated NH2-terminal domain of gi1alpha is sufficient to target a green fluorescent protein reporter to caveolin-enriched plasma membrane domains. Palmitoylation of caveolin-1 is required for the recognition of dually acylated g-protein alpha subunits in vivo. J Biol Chem 274: 5843–5850. 10026207 10.1074/jbc.274.9.5843

[29] Lee H, Woodman SE, Engelman JA, Volonte D, Galbiati F, et al (2001) Palmitoylation of caveolin-1 at a single site (Cys-156) controls its coupling to the c-Src tyrosine kinase: targeting of dually acylated molecules (GPI-linked, transmembrane, or cytoplasmic) to caveolae effectively uncouples c-Src and caveolin-1 (TYR-14). J Biol Chem 276: 35150–35158. 11451957 10.1074/jbc.M104530200

